# Antimicrobial Effect and Cytotoxic Evaluation of Mg-Doped Hydroxyapatite Functionalized with Au-Nano Rods

**DOI:** 10.3390/molecules26041099

**Published:** 2021-02-19

**Authors:** Domenico Franco, Giovanna Calabrese, Salvatore Petralia, Giulia Neri, Carmelo Corsaro, Lucia Forte, Stefano Squarzoni, Salvatore Guglielmino, Francesco Traina, Enza Fazio, Sabrina Conoci

**Affiliations:** 1 Department of Chemical, Biological, Pharmaceutical and Environmental Sciences, University of Messina, 98166 Messina, Italy; dfranco@unime.it (D.F.); giovanna.calabrese@unime.it (G.C.); giulia.neri@unime.it (G.N.); sguglielmino@unime.it (S.G.); 2 Department of Drug Science and Health, University of Catania, 95125 Catania, Italy; salvatore.petralia@unict.it; 3 Department of Mathematical and Computational Sciences, Physics Science and Earth Science, University of Messina, 98166 Messina, Italy; carmelo.corsaro@unime.it (C.C.); enfazio@unime.it (E.F.); 4 Fin-Ceramica Faenza, 48018 Faenza, Italy; lucia.forte@finceramica.it; 5 CNR-Institute of Molecular Genetics “Luigi Luca Cavalli-Sforza”, 40136 Bologna, Italy; squarzoni@area.bo.cnr.it; 6 IRCCS Istituto Ortopedico Rizzoli, 40136 Bologna, Italy; traina.francesco@gmail.com; 7 Department of Biomedical, Dental, Morphological and Functional Imaging Sciences, University of Messina, 98125 Messina, Italy

**Keywords:** bone tissue engineering, Au nanoparticles, hydroxyapatite, hybrid biomaterials, nano-functionalization, antibacterial activity, cytotoxicity

## Abstract

Hydroxyapatite (HA) is the main inorganic mineral that constitutes bone matrix and represents the most used biomaterial for bone regeneration. Over the years, it has been demonstrated that HA exhibits good biocompatibility, osteoconductivity, and osteoinductivity both in vitro and in vivo, and can be prepared by synthetic and natural sources via easy fabrication strategies. However, its low antibacterial property and its fragile nature restricts its usage for bone graft applications. In this study we functionalized a MgHA scaffold with gold nanorods (AuNRs) and evaluated its antibacterial effect against *S. aureus* and *E. coli* in both suspension and adhesion and its cytotoxicity over time (1 to 24 days). Results show that the AuNRs nano-functionalization improves the antibacterial activity with 100% bacterial reduction after 24 h. The toxicity study, however, indicates a 4.38-fold cell number decrease at 24 days. Although further optimization on nano-functionalization process are needed for cytotoxicity, these data indicated that Au-NRs nano-functionalization is a very promising method for improving the antibacterial properties of HA.

## 1. Introduction

Bone tissue engineering (BTE) requires the use of biomaterials with adequate mechanical properties and mostly with specific features able to mimic the original tissue structure.

Furthermore, the biomaterials must also provide, beyond biocompatibility, osteoconductivity, and osteoinductivity, a number of other factors such as good degradation rate, porosity, and surface characteristics, to improve cell adhesion, migration, proliferation, and differentiation [1,2]. HA is one of the most used biomaterials for bone regeneration because its inorganic component is similar to natural apatite, the mineral that constitutes bone matrix and provides rigidity to bone [3,4]. It is biocompatible, minimally inflammatory, osteoconductive, osteoinductive, and biodegradable [5,6]. Moreover, HA can be prepared both by synthetic and natural sources via easy fabrication strategies, which makes it an attractive grafting material [7]. Although HA shows these interesting properties, recent studies have shown that it alone is not suitable to be employed as an implant for load-bearing applications, mainly due to its low antibacterial property, one of the most important clinical needs for BTE, and also for its structural fragility [8].

Therefore, some efforts are still necessary to obtain HA-based materials that are mechanically strong, chemically inert, adequately biodegradable, and especially capable of offering a good antibacterial activity.

Recently, several evidences focused on HA scaffold matrix functionalization with nanoparticles (NPs), including metal NPs, to improve physical–chemical, mechanical, and biological properties [9]. Nanotechnology is a powerful tool for improving material properties [10]. Metal NPs have attracted great interest for some their innate features such as their mechanical strength, capability to stimulate osteogenic and angiogenic activities, potential antimicrobial properties, and finally their photosensitive properties, which can be tuned through the changing of particle size and shape [11,12]. Several metal and metal-oxide NPs with antimicrobial activity have been studied and identified, such as silver (Ag), silver oxide (Ag_2_O), zinc (Zn), zinc oxide (ZnO), titanium (Ti), titanium dioxide (TiO_2_), copper (Cu), copper oxide (CuO), and gold (Au) [13]. The addition of metal NPs is also considered an appropriate strategy in terms of guiding the tissue response in contact with specific stimuli (i.e., light irradiation). Among them, AuNPs represent interesting materials due to their multiple properties, such as photothermic properties [14,15,16], and their ability to act as X-ray contrast agents [17], probes for the detection of biomolecules, [18] etc. All these uses give rise to surface plasmon resonance ability, surface-enhanced Raman scattering (SERS) properties, and the fluorescence enhancement or quenching capacity of AuNPs [19,20]. Moreover, it has been reported that Au nanostructures exhibit osteoinductive and cytotoxic activities strictly correlated to the size, shape, concentration of NPs, and cell line tested [21,22,23]. However, a deep control of these properties is needed to improve the performance of Au nanostructures in bone regeneration therapy.

Thus, the combination of Au nanostructures with HA materials stimulated the design of a new scaffold with multiple properties, optimizing antimicrobial and chemical properties without any degradation of the HA porosity.

In this study we developed a new gold nanorod (AuNR)_MgHA scaffold, obtained by surface functionalization of MgHA with AuNRs, and evaluated its antibacterial effect against *S. aureus* and *E. coli* in both suspension and adhesion, and its cytotoxicity.

## 2. Results and Discussion

### 2.1. AuNR_MgHA Scaffold

**Figure 1:**
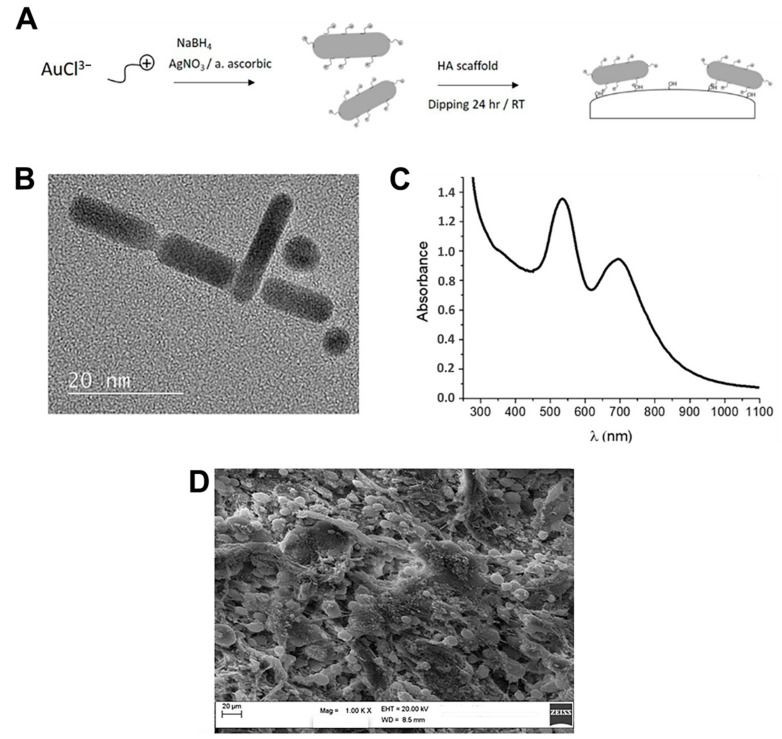
(**A**) Schematic reaction steps of AuNR_MgHA scaffold preparation; (**B**) Representative transmission electron microscopy (TEM) image of AuNR nanostructures; (**C**) UV optical absorption spectrum of AuNR dispersion; (**D**) SEM image of AuNR_MgHA scaffold. Magnification 1000×.

The AuNRs were synthetized according to the process described by Calabrese et al. [24] and are schemed in Figure 1A. The structural features of the AuNRs, prior to the surface functionalization of the MgHA scaffold, were analyzed by transmission electron microscopy (TEM) analysis. Figure 1B reports the TEM images showing spherical structures of AuNRs characterized by a diameter of ~5 nm and ~18 nm in length (aspect ratio ~3.6). The characteristic UV absorption spectrum (Figure 1C) shows two main features centered at ~530 nm and 730 nm, respectively. The position and intensity of these peaks are strongly influenced as function of the value of aspect ratio, but in case of Au nanostructures of diameters less than 25 nm this influence is negligible [25,26]. After the MgHA scaffold surface functionalization with AuNRs, we performed a morphological and microstructural analysis by scanning electron microscopy (SEM). The investigation highlights a well interconnected porous structure with a granular and rough surface, without any favored alignment of the collagen fibers (Figure 1D).

### 2.2. Antibacterial Activity of the AuNR_MgHA Scaffold

**Figure 2:**
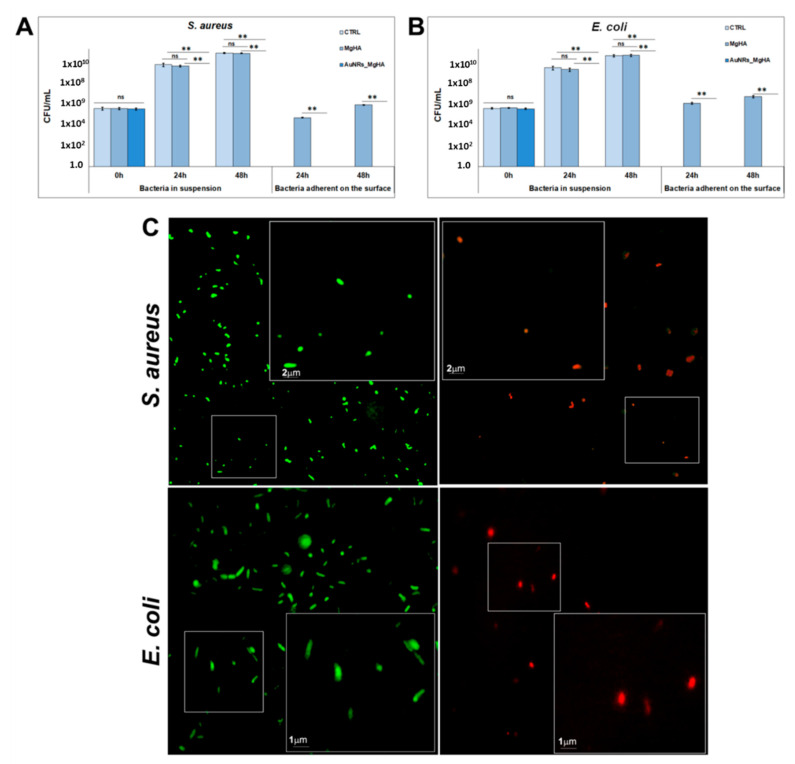
(**A**,**B**) Number of colonies (CFU) in MgHA and AuNR_MgHA scaffolds after 24 and 48 h of incubation for *S. aureus* and *E. coli*, respectively. The mean and standard deviation were obtained from three replicates. ANOVA test *p* values were reported (*p* < 0.0001) and (** *p* < 0.01) indicating significant differences between scaffolds as reported by the Tukey post-hoc test; ns = not significant. (**C**) Live/dead staining of *S. aureus* and *E. coli*, on MgHA and AuNR_MgHA scaffolds. The inserts are magnifications of traced areas.

The antibacterial properties of AuNR_MgHA scaffolds were tested against *Staphylococcus aureus* (*S. aureus*) and *Escherichia coli* (*E. coli*), both in suspension and in adhesion (Figure 2A,B). Data showed a complete loss of bacteria, in both conditions, after 24 h in AuNR_MgHA scaffolds compared to the control (MgHA). Specifically, the colony number in MgHA scaffolds was (2.6 ± 0.5) × 10^9^ at 24 h and (4.0 ± 0.4) × 10^10^ at 48 h for *S. aureus,* (1.2 ± 0.4) × 10^9^ at 24 h and (2.6 ± 0.6) × 10^10^ at 48 h for *E. coli,* in suspension; whereas, in AuNR_MgHA scaffolds for both bacteria no colonies were found after 24 h. The number of suspended bacterial cells alone was similar to that of bacteria grown on MgHA scaffolds, (3.4 ± 1.0) × 10^9^ and (4.2 ± 0.5) × 10^10^ CFU/mL at 24 h and 48 h, respectively, for *S. aureus*, and (1.8 ± 0.7) × 10^9^ at 24 h and (2.4 ± 0.6) × 10^10^ at 48 h for *E. coli.*

Similar results were obtained in adhesion on the scaffold surface for both bacteria. In further detail, the colony number in MgHA scaffolds was (3.8 ± 0.4) × 10^4^ at 24 h and (5.9 ± 0.7) × 10^5^ at 48 h for *S. aureus,* and (8.7 ± 1.1) × 10^5^ at 24 h and (3.4 ± 0.8) × 10^6^ at 48 h for *E. coli*. On the other hand, in AuNR_MgHA scaffolds no colonies were found at both time points for both bacteria (Figure 2A,B).

These results were in agreement with the live/dead staining (Figure 2B) which showed that MgHA did not affect bacterial viability, as evidenced by the presence of a large number of green fluorescent living cells (Syto9); in contrast, most cells are dead in the AuNR_MgHA scaffold, as indicated by red fluorescence (propidium iodide).

In term of bactericidal mechanism, many works have reported on the significant antibacterial (against both Gram+ and Gram–) and antifungal activity of AuNPs [27]. On the other hand, the cytotoxic activity of AuNPs can also depend on their size, surface functionalization, and aggregation phenomena that prevent endocytic processes [28,29]. In the latter case, although AuNPs with smaller sizes aggregate with more difficulty, growth media could affect their colloidal stability. The presence of the MgHA scaffolds could help stabilize the AuNRs and favor the endocytic processes mediated by bacterial cells. Once inside the cells, the bactericidal effect is carried out through blocking the expression of some genes, inhibiting ATP synthesis, and consequently dissipating membrane potential, producing ROSs [30].

### 2.3. Evaluation of AuNR_MgHA Scaffold Cytotoxicity

**Figure 3:**
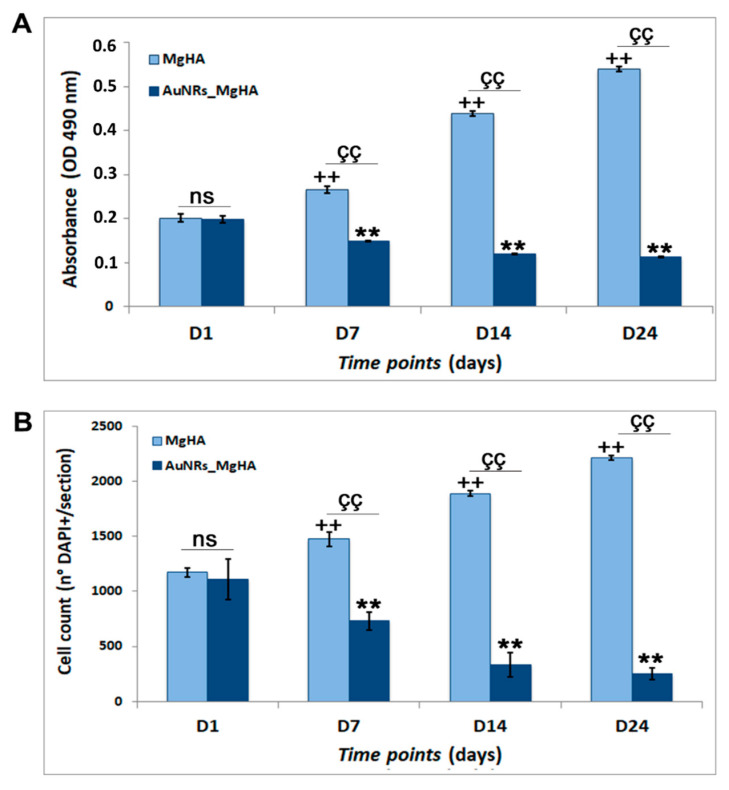
(**A**) Cell viability (MTS assay) after 1, 7, 14, and 24 days of hADSCs culture on MgHA and AuNR_MgHA scaffolds. (**B**) Cell count from DAPI staining at the four different timepoints of the culture. The ANOVA test *p* values reported *(p* < 0.0001) and (**, çç, ++ *p* < 0.01) and *(p >* 0.05 ns = not significant) indicate significant differences between scaffolds, as reported by the Tukey post-hoc test.

In order to evaluate the cytotoxicity of the AuNR_MgHA scaffolds, we cultured hADSCs on the scaffold surfaces and we analyzed their ability to grow within them at four specific time points (1, 7, 14, and 24 days) by MTS assay and DAPI staining. Data are reported in Figure 3. Results show that the cell number in the scaffolds decreases drastically over time, from the 1^st^ to the 24^th^ day, in a statistically significant manner (*p* < 0.01). In further detail, the MTS results of the AuNR_MgHA scaffolds show that cell viability decreased about 1.02-fold at day 1, 1.8-fold at day 7, 4.0-fold at day 14, and 5.2-fold at day 24, compared to the MgHA scaffolds (Figure 3A). These data were further confirmed by nuclei counts through DAPI staining (Figure 3B), displaying that the cell amount in the AuNR_MgHA scaffolds decreased about 1.06-fold at day 1, 2.02-fold at day 7, 5.69-fold at day 14, and 8.73-fold at day 24, compared to the MgHA scaffolds.

These results suggest that the AuNR_MgHA scaffolds are not able to stimulate hADSCs proliferation, indeed they reduce their growth probably due to their cytotoxicity. These data are consistent with a lot of in vitro studies on AuNP cytotoxicity, even though the mechanism of this is not yet clear [31]. In fact, although AuNPs are widely used for biomedical applications, their cytotoxic and biological effects in several cell lines are often contradictory. Tang et al. [32] correlated the cytotoxicity of AuNRs to their concentration and induced-oxidative stress. Further, Chuang et al. reported that AuNRs are cytotoxic to most mammalian cells, but their effect depends primarily on the cell type rather than on the size and cellular uptake [33].

**Figure 4:**
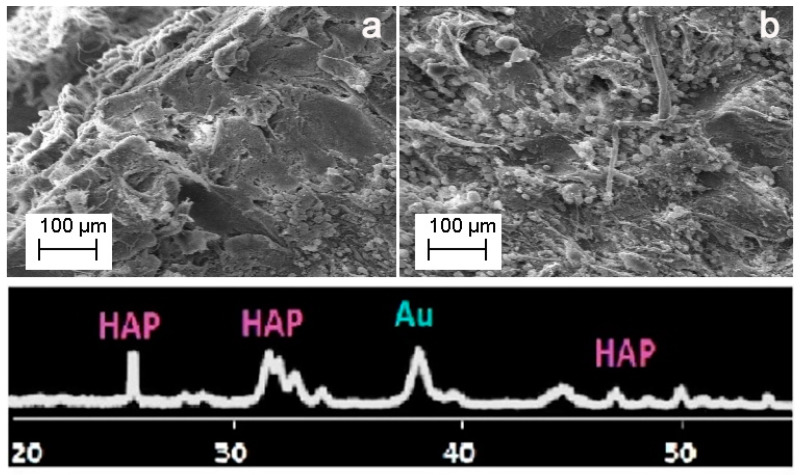
Representative SEM images of AuNR_MgHA scaffolds without (**a**) and with cells (**b**), and XRD analysis at day 7. Magnification 200×.

The morphology of AuNR_MgHA with cells was analyzed by SEM. In Figure 4 shows a representative SEM image and an X-ray diffraction (XRD) spectrum of the AuNR_MgHA scaffolds with the cells after 7 days of culture. It can be noticed that the AuNR_MgHA scaffold with cells exhibits a similar morphological profile to the AuNR_MgHA scaffold without cells. XRD analysis confirmed that AuNR_MgHA displays a typical phase composition of MgHA scaffold. The (002), (211), (112), (300), (130), (222), (213), and (004) planes of the hexagonal HA unit cell were recorded [34]. Furthermore, the most intense peak detected at about 2θ~38° is ascribed to the metallic Au, according to the JCPDS no. 071-4614.

Since the surface chemical properties of implanted materials are key features in their biological response, high-sensitive surface techniques are required for their characterization. X-ray photoelectron spectroscopy (XPS) is one of these techniques, which is widely employed for qualitative and quantitative analysis of biomaterials, probing the outermost atomic monolayers of their surface [35,36]. The chemical species in our samples exposed to cells and their content, expressed as atomic percentages considering the relative atomic Scofield’s sensitivity factors, are: P (~8.0%), Ca (12%), C (20.0%), N (2.0%), O (56%), Au (1.5%), and Mg (0.5%), respectively. The AuNR_MgHA scaffolds are nearly stoichiometric with a Ca/P value of 1.8 and 1.7, against 1.6 of the MgHA control. Further, the O/Ca, O/P, and C/Ca values are almost higher with respect to the control. However, some oxygen atoms are also bonded to nitrogen and carbon atoms, as indicated by N1s and C1s lineshapes. Nitrogen species are ascribed to the contribution of peptides from cells and/or N–C bonds, as these samples contained amine groups cross-linked with AuNR_MgHA [19].

**Figure 5:**
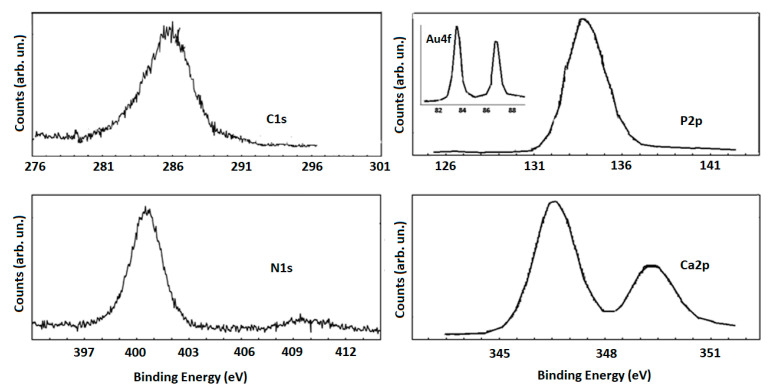
High-resolution XPS spectra of C1s, P2p, N1s, and Ca2p lineshapes.

XPS representative high-resolution spectra are reported in Figure 5. The Ca2p3/2 peak at about 347 eV is typical of the presence of Ca in a hydroxyapatite phase, in the same way that the P2p peak centered at about 134.0 eV is due to a CaPO_4_ phase [37].

These XPS profiles are relatively broad, because of the presence of a further oxide phase for P2p peaks and the presence of carbonates for the Ca2p peaks (http://www.xpsfitting.com/search/label/Phosphorus, accessed on 17 February 2021). Furthermore, the C1s peak is characterized by specific features: the band at about 285 eV could be attributed to aliphatic C–C carbons of the amino acid side chains, the band located at about 285.5 eV could be due to C–N carbons of the main peptide/lysine side chains, while the peak located at 287.4 eV could arise from the N–C=O peptide bond and to O–C=O carbons of the glutamate side chains [38]. These attributions are consistent to those referred to in the N1s spectrum where the main signal at about 400 eV is ascribed to the unprotonated nitrogen atoms of the peptide backbone, while the lower intensity feature at about 401.9 eV is generally attributed to the protonated nitrogen atoms of the lysine pending groups, that pair with the carboxylate groups of glutamate by ionic bonds [39]. Finally, the Au XPS spectrum, shown in the inset of Figure 5, confirms the metallic nature of AuNRs.

The evidences described above highlight that, despite the AuNRs derivatization conferring excellent antibacterial activity, some weaknesses are still present for in vitro biocompatibility suggesting some limitations in their use for implantation in vivo. Future studies will be devoted to address these limitations by improving the biocompatibility.

## 3. Materials and Methods

### 3.1. Preparation of the AuNR_MgHA Scaffold

The AuNR_MgHA scaffold was obtained by a synthetic process reported in [24]. AuNRs were prepared via a chemical reduction strategy, by using CTAB (hexadecyltrimethylammonium bromide) as a surfactant agent. Briefly, HAuCl_4_ solution was heated up to 75 °C and an equal volume of a CTAB solution was added. Afterwards, AgNO_3_ solution, L-ascorbic acid solution, and NaBH_4_ were also added and the reaction mixture was stirred in darkness at room temperature for 30 min. Then, AuNRs were collected by centrifugation technique and washed with deionized water. The MgHA scaffolds used in this study were provided by Fin-Ceramica Faenza SpA (Faenza-Italy) and produced according to the process described in [5,40]. To prepare AuNR_MgHA, MgHA scaffold was first immersed in an AuNRs aqueous solution overnight, then washed with deionized water and finally dried under N2 flow.

Transmission electron microscopy (TEM) analysis was performed using the bright field in conventional TEM parallel beam mode. An ATEM JEOL JEM-2010 (JEOL Ltd., Musashino, Akishima, Tokyo, Japan), equipped with a 30 mm^2^ window energy-dispersive X-ray (EDX) spectrometer, was used. UV–Vis optical absorption spectra were recorded with a Jasco V-560 homemade photoreactor equipped with UV light (four lamps; 16 Watt, 254 nm).

Morphological analyses were acquired both by a Philips XL-30-FEI SEM (Philips, Amsterdam, The Netherlands) and a Zeiss EVO MA10 (Zeiss international, Headquartered in Oberkochen, Germany) equipped with an LaB6 electron gun, at the operating voltage of 20 kV. Phases and structures present in the synthesized samples were investigated by X-ray diffraction (XRD) analysis. XRD patterns were recorded, in the 2θ range from 20° to 80°, using a Bruker D8 advance X-ray diffractometer (Bruker, Billerica, MA, USA) with Cu Kα radiation (1.5406 Angstrom).

The chemical composition and the bonding configurations of the scaffolds were investigated by means of X-ray photoelectron spectroscopy (XPS). The spectra were acquired at room temperature using a Thermo Scientific K-Alpha system, equipped with a monochromatic Al Kαsource (1486.6 eV) and operating with a hemispherical analyzer operating in constant-pass energy (CAE) mode. The pass-energy was set at 200 eV for survey scans and at 50 eV for the XPS core-level spectra. A spot size diameter of about 400 μm was adopted while a charge neutralizer (low energy e-beam 0.2 V at 100 µA) was used for charge compensation. Binding energy shifts were calibrated keeping the C1s position fixed at 284.5 eV.

### 3.2. Antibacterial Activity of the AuNR_MgHA Scaffold

*S. aureus* (ATCC29213) and *E. coli* (ATCC19138) were purchased from the American Type Culture Collection (LGC Promochem, Milan, Italy), cultured in Tryptone Soya Broth (TSB, Sigma-Aldrich, Milan, Italy) and Luria-Bertani Broth (LB, Sigma-Aldrich, Milan, Italy), respectively, and maintained in 20% glycerol at −80 °C.

All scaffolds were decontaminated before being used, through the use of a UV lamp for 12 h.

Bacterial semi-exponential cultures were used for all bactericidal assays. Specifically, *S. aureus* and *E. coli* overnight cultures were inoculated in fresh medium and incubated for 6–8 h at 37 °C under shaking at 150 rpm. Then bacteria were centrifugated and resuspended in phosphate-buffered saline (PBS) three times. Starting from this bacterial solution having an equivalent turbidity (McFarland standard) of 0.5 (approximately 2 × 10^8^ CFU/mL), serial dilutions were made to finally obtain 2 × 10^5^ bacteria/mL in TSB (Tryptic Soy Broth) medium.

Bacteria suspension was added to the pre-decontaminated scaffolds in 24-well plates (1 mL/well), and incubated at 37 °C under gentle shaking (100 rpm, orbital shaker KS-15, Edmund Bühler GmbH). MgHA scaffolds were used as positive control of normal bacterial growth.

The antibacterial effect of the scaffolds was evaluated both in suspension and in adhesion, after 24 and 48 h of incubation by Colony Forming Units (CFU) assay. For bacteria in adhesion, the scaffolds incubated with *S. aureus* and *E. coli* (after 24 h and 48 h) were placed in a new well, washed three times in PBS to remove non-adherent bacteria, placed in a glass tube containing 1 mL of PBS, and sonicated for 60 s in an ultrasonic bath (frequency 35 KHz). After, bacteria suspensions were diluted 1:10 in PBS and 100 μL of each dilution were spread on agar medium and incubated overnight at 37 °C. Plates in the range of 30–300 colonies were evaluated for CFU count by the following Equation (1):

(1) $$CFU=\frac{number\;of\;colonies}{\left(volume\;\left(0.1\;mL\right)\;\times dilution\;factor\right)}$$

Live/dead BacLight bacterial viability assay was also performed as follows: 6 μL of dye, SYTO 9 and propidium iodide (1:1), were added to 200 µl of bacterial suspension and incubated for 15 min at 37 °C [41]. The samples were analyzed using a Leica DMRE epifluorescence microscope with Leica C Plan 63× objective and BP 515–560 nm excitation filter combined with a LP 590 nm suppression filter.

### 3.3. Cytotoxic Analysis of the AuNR_MgHA Scaffold

The cell line hADSCs (Thermo Fisher Scientific, NYSE:TMO) used in this study has been derived from human lipoaspirate tissue and cryopreserved from primary cultures. hADSCs were chosen because they give rise to bone cells under appropriate conditions, as we previously demonstrated, both in vitro and in vivo [40,42]. The cells were cultured in a complete culture medium, MesenPRO RSTM (Thermo Fisher Scientific, NYSE:TMO), and maintained in a humidified environment at 37 °C and 5% CO_2_ until about 70–80% confluence was achieved. The hADSCs at the third passage (P3) were used for the following experiments.

1 × 10^6^ cells diluted in 50 μL of culture medium were slowly seeded onto the scaffold surfaces, placed in 24-well plates, and incubated at 37 °C for 4 h. After 4 h, the culture medium was added until the scaffolds were completely covered and maintained in culture for 24 days. The medium was wholly substituted twice a week, and the scaffolds analyzed at specific time points (1, 7, 14, and 24 days) through DAPI (4′,6-diamidino-2-phenylindole) staining.

For MTS assay we added the MTS solution to the cell culture media and incubated for 2 h in standard culture conditions. After briefly shaking the plate, the absorbance at 490 nm was read using a synergy HT plate reader (BioTek Instruments, Inc., Winooski, VT, USA).

DAPI staining was performed as follows. hADSCs-AuNR_MgHA scaffolds, at several time points, were fixed in 4% PFA, dehydrated, embedded in paraffin, and cut into 5 μm-thick sections. Sections were permeabilized in 0.3% Triton X-100 for 10 min, washed in phosphate-buffered saline (PBS), and the nuclei stained with DAPI (1:10,000) in PBS for 5 min. Successively, the slides were mounted, with fluorescent mounting medium (PermaFluor, Thermo Scientific, Waltham, MA, USA), and digital images were acquired using a Leica DMI4000B fluorescence microscope. Four sections from each scaffold were analyzed and the nuclei counted using Fiji image J recognition software.

### 3.4. Statistical Analysis

Data were analyzed either as raw data or as mean ± standard error (SE), as appropriate. Differences between several time points of the hADSCs-AuNR_MgHA scaffolds were evaluated by using one-way ANOVA with a post-hoc Tukey HSD test, where appropriate. *p* < 0.05 was considered to be significant.

## 4. Conclusions

In this work we developed an AuNR_MgHA scaffold, through the surface functionalization of MgHA with AuNRs, and tested it for both its antibacterial effect against *S. aureus* and *E. coli* and its cytotoxicity over time (1 to 24 days). Our findings show that AuNR_MgHA scaffolds are already able to reduce the bacteria both in suspension and adhesion by 100% after 24 h, are showing good antibacterial properties against both bacterial strains. On the contrary, the cytotoxicity results demonstrated a noticeable cell number reduction over time, with a 4.38-fold decrease demonstrated from day 1 to day 24, suggesting that AuNR functionalization induces a cytotoxic effect on hADSCs. The cytotoxicity findings are in accordance with some previous literature evidences which report on how the toxicity of AuNRs on different cell models may be due to several factors, such as size, shape, surface area/porosity concentration, surface charges, aggregation, surface modifications, and host–cell interactions. In the light of this evidence and concerning the relevant antibacterial activity of AuNR_MgHA scaffolds, our future studies will be directed towards better understanding the AuNR cytotoxicity mechanism. Therefore, AuNRs of different sizes, shapes, concentrations, and surface modifications will be tested on several cell lines in order to improve their biocompatibility. Additionally, it will be important to understand the long term behavior of AuNPs in vivo before their application in BTE.

## Data Availability

Not applicable.

## Abbreviations

AuNRs	gold nanorods
BTE	bone tissue engineering
CAE	constant-pass energy
CTAB	hexadecyltrimethylammonium bromide
CFU	colony forming units
EDX	energy-dispersive X-rays
HA	hydroxyapatite
NPs	nanoparticles
PBS	phosphate-buffered saline
SEM	scanning electron microscopy
TEM	transmission electron microscopy
TSB	tryptone soya broth
XPS	X-ray photoelectron spectroscopy
XRD	X-ray powder diffraction
